# The effect of skin-to-skin contact at birth, early versus immediate, on the duration of exclusive human lactancy in full-term newborns treated at the Clínica Universidad de La Sabana: study protocol for a randomized clinical trial

**DOI:** 10.1186/s13063-016-1587-7

**Published:** 2016-10-26

**Authors:** Sergio Agudelo, Oscar Gamboa, Fabio Rodríguez, Sandra Cala, Nathalie Gualdrón, Evelyn Obando, María Lucía Padrón

**Affiliations:** 1 Universidad de La Sabana, Clínica Universidad de La Sabana, Chía, Cundinamarca Colombia; 2 Assistant professor Universidad de La Sabana, Neonatal Care Unit Coordinator of Clínica Universidad de La Sabana, Campus Puente del Común, Km. 7 Autopista al Norte de Bogotá, 53753 Chía, Cundinamarca Colombia; 3 School of Medicine, Universidad de la Sabana, Campus Puente del Común, Km. 7 Autopista al Norte de Bogotá, Chía, Cundinamarca Colombia

**Keywords:** Breast feeding, Skin-to-skin contact

## Abstract

**Background:**

Human lactancy is a simple and cost-effective strategy that influences infant and maternal mortality rates. Skin-to-skin contact (SSC) is an immediate postpartum period strategy that has proven to benefit the initiation and continuation of human lactation and to decrease hospitalization during the first week of life. This study aims to determine the effect of SSC initiation at birth (immediate versus early) in healthy, full-term newborns treated at the Universidad de La Sabana Clinic on the duration of exclusive human lactation.

**Methods/design:**

A randomized, blind clinical trial will be performed with full-term healthy newborns born at the Universidad de La Sabana Clinic. The blind trial participants will be those persons measuring the results and analyzing the data. The sample size will be calculated for a type I error of 5 %, a two-tailed type II error of 20 %, and an estimated percentage loss of 30 %; 150 infants will be included in each group. Randomization will be performed using permuted, size-6 blocks. Descriptive analysis will be conducted using central tendency and dispersion measurements. A bivariate analysis will be performed to determine which variables are associated with exclusive lactancy at 6 months. For continuous variables, Student’s *t* test will be used for independent samples, and the Wilcoxon rank sum test will be used if the assumptions of normality for the *t* tests are not fulfilled. The assumption of normality will be evaluated using the Shapiro-Wilk and Kolmogorov-Smirnov tests. Categorical variables in contingency tables will be constructed to assess the independence between variables using the chi-square test, or Fisher’s exact test when the assumption of the number of cases is not met by the values in the contingency tables multiplied by two. This will be calculated as a measurement of the effect of relative risk (RR) with confidence intervals; the adjusted measurements will be calculated using a multivariate regression Poisson model. Variables with significant results will be used in the bivariate analysis, and those with biological plausibility will be used for the adjustment. The analysis will be carried out for a two-tailed type I error level of 5 %. The Stata 11 program will be used for data analysis. An interim analysis will be performed upon the submission of half the expected events (106), setting limits for the early termination of the trial according to the method proposed by Pampallona and Tsiatis (1994). Intervention: There will be two SSC randomization groups: early versus immediate. After completing the neonatal adaptation process and based on the group assignment, the mother will be left with her newborn child in hospital accommodation. Prior to discharge, the Infant Breast-Feeding Assessment Tool (IBFAT) will be applied. Monitoring will initially be performed with a face-to-face assessment between 3 and 10 days of life, followed by monthly telephone calls for 6 months to verify lactation status.

**Discussion:**

SSC at birth has shown benefits in the short and long term for both the mother and the full-term newborn. Although the meta-analysis that have been done have shown the benefits of this technique, multiple differences in the SSC interventions have been identified because criteria such as the initiation or duration of SSC (dose) have not been unified. Colombia has a malnutrition risk of 11,4 % in the total population for the period 2012-2014, so it is necessary to promote strategies that generate a positive impact on the duration of human lactation, providing support from the clinical setting of humanized delivery which is included in the IAMI strategy (Instituciones Amigas de la Mujer y la Infancia – Friends of Women and Children Institution). Therefore, we propose that the initiation time of SSC in full-term new-borns is related to the duration of exclusive human lactation.

**Trial registration:**

Registered ClinicalTrials.gov Identifier: NCT 02687685. Registered on 2 February 2016. This study is not yet open for participant recruitment.

**Electronic supplementary material:**

The online version of this article (doi:10.1186/s13063-016-1587-7) contains supplementary material, which is available to authorized users.

## Background

### Scientific background and justification

The first 2 h of a baby’s life has been defined as the optimum time to begin human lactation. Skin-to-skin contact (SSC) between newborn and mother in this period improves the chance of successful lactancy in the first hour of life and in the long term [1, 2]. The exclusive use of breast milk is the optimal food for infants in the first 6 months. The SSC birth involves placing the naked newborn in the prone position on the bare chest of the mother, drying and covering the baby’s head with a cap and the baby and mother with a sheet. This strategy offers benefits to the mother and baby in both the short and long term. Secondary complications for the mother (e.g., mastitis) often lead to inadequate breastfeeding whereas adequate breastfeeding decreases rates of maternal postpartum bleeding and depression, leads to greater cardiorespiratory and thermal stability of the newborn and a decrease in the need for newborn hospitalization within the first week of life; it also initiates the colonization of the mother’s own gut flora in the newborn which leads to a decreased risk of infection [3–5], a lower incidence of infectious diseases in the first year of life, and a positive impact on reducing the risk of obesity and of contracting chronic noncommunicable diseases; these human lactation benefits are directly related to the duration of lactation [6, 7].

The SSC strategy has been applied at different times at birth and for different time durations, the latter being understood as being a dose of SSC. Depending on the time of initiation of the SSC intervention, it has been divided into subcategories (Table 1) [3]. Although the optimum duration for SSC has not been defined, it has been accepted that we must continue and maintain it at least until the baby has completed its first proper sucking of the breast and is physiologically stable [1, 3, 6]. In Colombia, in the 2013 Clinical Practice Guidelines of the Ministry of Health and Social Protection for healthy newborn care, it is recommended that SSC should be made immediately during the postpartum procedure while performing immediate interventions and postponing newborn adaptation measures in order to prevent the separation of the newborn from their mother in the immediate newborn period [8]. In the obstetric literature, it is not clear if the benefits of the strategy remain independent of the initiation time of SSC at birth [3]. In Colombia however, the average duration of exclusive lactancy is 1.8 months which is well below the worldwide recommendation; additionally, only 56.6 % of Colombian mothers feed their babies breast milk in the first hour of life [9]. Currently, widespread care practices for mothers and newborns in hospitals and care centers make it common for the mother and baby to be separated in the immediate moment following birth, breaking the bonding link between them at this time and altering the ratio and benefits deriving from this contact [10, 11].

**Table 1:** Subcategories on the time of initiation of skin-to-skin contact (SSC) intervention

At birth or immediately	Very early	Early
When contact is made within the first minute of birth	Within the first 30 to 40 min after birth and after the mediate and immediate neonatal adaptation interventions have been carried out	At any time between the first hour and 24 h of life

Through conducting a randomized, blind clinical trial, this study aims to determine the effects of two different SSC initiation times (immediate versus early) on the duration of exclusive human lactation in healthy full-term newborns treated at the Universidad de La Sabana Clinic.

### State of the art

Variability is evident in both clinical practice and the obstetric literature for both the optimum start time for implementing SCC and its optimum duration, which has created heterogeneous strategy results in research studies as well as interpretation. No studies have been conducted comparing the different SSC initiation times regarding breastfeeding, while recommendations have been made to study whether the initiation time makes a difference in the benefits of the technique [3].

Interventions at birth and the practice of separating the baby from the mother at birth have negative effects on the initiation of breastfeeding. A cross-sectional study of the factors involved in the initiation of lactancy within the first hour of life was conducted in different hospitals in Rio de Janeiro between 1999 and 2001 [11], and found that human lactation occurring during the first hour of life was less frequent if infants were subjected to immediate interventions after birth (odds ratio (OR) 0.47, 95 % confidence interval (CI) 0.15 to 0.80), if mothers had no contact with their newborns in the delivery room (OR 0.62, 95 % CI 0.29 to 0.95), if mothers were undergoing caesarean section (OR 0.48, 95 % CI 0.24 to 0.72) and if mothers were treated at private institutions. Regarding practices that promote exclusive breastfeeding during the hospital stay postpartum, Bramson et al. [12] in California, USA, conducted a cohort study; the analysis of multivariate logistic regression showed that the following factors – intention of breastfeeding before birth, the sociodemographic characteristics of the mother and early SSC applied within the first 3 h of the birth – all correlated positively with exclusive human lactancy during the hospital stay. In addition, a link between the duration of the SSC (dose) and exclusive human lactancy was revealed and there is a directly proportional relationship between the dose or time and exclusive lactancy: contact time between 1 and 15 min (OR 1.37, 95 % CI 1189 to 1593), 16 and 30 min (OR 1.66, 95 % CI 1468 to 1888), 31 and 59 min (OR 2.35, 95 % CI 2061 to 2695), and more than an hour (OR 3.14, 95 % CI 2905 to 3405).

Carfoot et al. [13] conducted a randomized clinical trial in the UK in order to assess the effects of early SSC in healthy newborns over 36 weeks on the initiation and duration of human lactation. It included 204 mothers and their newborns who were divided randomly into two groups: early SSC (*n* = 102): initiated at birth, delaying the adaptation intervention until after contact, the duration was at least 45 min until the first feed was completed or the mother chose to withdraw the SSC and the control group, were receiving routine care (*n* = 102) understanding this to mean that once born, the routine adaptation interventions are applied, separating the newborn from the mother and/or parent. The investigators measured the success of the first breastfeed as a primary result and as secondary results they measured exclusive lactancy until 4 months, thermoregulation in the first hour of life and the mother’s degree of satisfaction. The IBFAT (Infant Breast feeding Assessment Tool) scale was used to evaluate the success of the baby’s first breastfeed. They found that the success of the first feed within the first hour of life was higher in the SSC group (mean 8 %, 95 % CI 1.6 % to 17.6 %) and also higher for the duration of exclusive breastfeeding until the first 4 months of life. Likewise, the thermal stability of the baby was better in the SSC group and mothers also reported greater satisfaction in this group.

Villalón et al. [14] in Chile, conducted a prospective randomized study with newborns of between 38 and 42 weeks’ gestation and weighing between 2500 and 4250 g at birth; the intervention group was defined as *early SSC* in which the baby and mother have contact at birth and SCC was continued for 4 h. The control group was defined as the *post-birth routine care* group in which the mother is separated from the newborn in order for adaptation care routines to be applied. They evaluated lactancy independent of whether suction took place at birth or not and they considered 2 to 4 h as adequate time for exclusive lactancy and inadequate for those requiring whole or mixed formula feeding. Significant differences were found in favor of the group using early SSC in exclusive breastfeeding at 24 h of life (89.9 % versus 63.3 %, *p* < 0.001), at discharge from hospital (93.3 % versus 66.7 %, *p* < 0.001), and at 14 days of age (78.8 % versus 56.2 %, *p* < 0.02).

The systematic review by the Cochrane Collaboration [3] found that the SSC has positive effects on human lactation during the first to fourth months of life (OR 1.82, 95 % CI 1.08 to 3.07) and a positive effect on the duration of lactancy (mean difference 42.55, 95 % CI −1.69 to −86.79). Trends in improvement in the overall scores of maternal affection during lactancy, as well as in maternal attachment behaviors, were also found. Other reported benefits are that the newborns cry less (mean difference −0.01, 95 % CI −8.98 to −7.04). No adverse effects were observed or reported. It is important to note that the reviewers reported limitations given the variability in the intervention (initiation time of contact and duration) and the definition of variables; proposing SSC initiation time subcategories (*immediate*, *birth*, *very early*, and *early*), thus highlighting the need for studies investigating the benefits of the technique in this field.

As to the time of SSC and its duration, Takahashi et al. [15] evaluated the effectiveness of the initiation time and the duration of the SSC using by three indices: the measurement of stress-related cortisol in saliva, circulatory evaluation of heart rate and respiratory adaptation, and oxygen saturation. They found that the body temperature at 60 min and 120 min was lower in the SSC *birth* group (*p* < 0.001) compared to the *very early group* (*p* < 0.05), but all remained within normal ranges. A faster heart rate stability was found in the SSC *birth* group compared to *early* (*p* = 0.001), there were no significant differences in respiratory adaptation. Stress levels as indicated by the cortisol level were lower in babies who were in SSC for more than 60 min compared to those who were in SSC for less than 60 min (*p* = 0.046). This study concludes that *early* SSC within the first 5 min of life, with a continuous duration of more than 60 min, reduces the stress on the baby and improves cardiopulmonary stability at birth.

In Iran, Aghdas et al. [16] evaluated the effect of immediate SSC efficacy for human lactation through a randomized clinical trial with first-time mothers, measured by the BSES (Breastfeeding Self-efficacy Scale) until day 28. The mothers were randomized into two groups: *early* SSC, in which the newborn was left in SSC with the mother from birth for 2 h, postponing interventions up until that point; and *routine care*, in which the baby was taken to the radiant heat lamp once the umbilical cord had been cut in order to proceed with the interventions. The success of the first breastfeed was evaluated as a secondary result using the IBFAT and the average first lactation. In the SSC group, the self-efficacy in human lactation (BSES) score was 53.42 (standard deviation (SD) 8.57) versus 49.85 (SD 5.5) in the control group (*p* = 0.0003). The successful initiation of human lactation was 56.6 % in the intervention group versus 35.6 % in the control group (*p* = 0.02) and finally, the initiation time of the first feed was 21.98 ± 9.1 min in the SSC group versus 66.55 ± 20.76 min in the routine care group (*p* = 0.001).

### Infant Breast-Feeding Assessment Tool (IBFAT)

Various tools or tests to assess lactancy have been developed. The IBFAT tool was developed and published by Matthews et al. in 1993 to evaluate the behavior of the baby during sucking and swallowing, with a reliability of 91 % [17]. Using six items, the behavior of the baby was quantified and evaluated during lactancy in the first week of life and the focus was concentrated on both the baby and the mother.

Schlomer et al. [18] evaluated two scores as tools to assess lactancy, to correlate problems during lactancy and the degree of maternal satisfaction. The LATCH tool, which is a system for the documentation of lactation, identifies areas where intervention is required to support lactancy and focuses on the role of the mother in the process of breastfeeding whereas the IBFAT scale focuses on the baby during feeding. They found that as the scores of both instruments increased there was a tendency to an increase in maternal satisfaction with a decrease in breastfeeding problems, but this was not statistically significant (LATCH *r* = 0.5, *p* = 0.06 and IBFAT *r* = 0.49, *p* = 0.06).

Riordan et al. [19] initially included the IBFAT, MBA (Mother-Baby ASSESS tool) and LATCH tools to assess the reliability and validity of three clinical assessment instruments for lactancy evaluation. They found that the coefficient reliability was not acceptable for clinical decision-making. Subsequently, Altuntas et al. [20] in 2104, again assessed the validity and reliability of these three scales, finding a positive and significant correlation; the MBA tool had a correlation ranging from 0.81 to 0.88, the IBFAT from 0.9 to 0.95, and the LATCH tool between 0.85 and 0.91. They concluded that the three scales or tools are compatible, reliable, and appropriate to evaluate the efficiency of lactancy.

### Objectives

To determine the effect of immediate versus early SSC birth on the duration of exclusive human lactancy in healthy, full-term newborns in the Universidad de La Sabana Clinic.

### Specific objectives

- To evaluate the competence of the newborn human lactancy in the first 24 h of life by using the IBFAT instrument among newborns in immediate SSC compared with early SSC
- To determine the prevalence for hospitalization and admission to the neonatal intensive care unit (NICU) in the first week of life between the two SSC groups (immediate versus early)

### Hypothesis

- ○ Null hypothesis: there is no difference in the percentage of healthy, full-term newborns receiving exclusive human breastfeeding for three or more months between the immediate versus early birth SCC groups: relative risk (*RR*) = 1
- ○ Alternative hypothesis: there is a difference in the percentage of healthy, full-term newborns receiving exclusive human breastfeeding for three or more months between the immediate versus early birth SCC groups: *RR* ≠ 1

## Methods/design

### Participants, interventions, and outcomes (see: Table 2 Schedule of enrolment, interventions, and assessments)

#### Participants

Included healthy, full-term newborns treated at the Universidad de La Sabana Clinic who meet the following criteria:

- ○ Inclusion criteria:
  - ▪ Infants of mothers who prenatally express their desire to breastfeed their newborn baby
  - ▪ Full-term newborns who are defined, by obstetric method and confirmed by pediatric method (Ballard), as being between 37 and 42 weeks of gestation with appropriate weight for gestational age (between 10^th^ and 90^th^ percentiles for gestational age)
  - ▪ Delivered by vaginal birth
  - ▪ Do not require basic or advanced neonatal resuscitation maneuvers
  - ▪ Have healthy and stable cardiorespiratory systems at birth
  - ▪ Have been permitted to room with the mother
- ○ Exclusion criteria:
  - ▪ Mothers and newborns who present absolute or relative contraindications for human lactancy
  - ▪ Multiple pregnancies and births
  - ▪ Mothers with postpartum complications that limit the onset of human lactation
  - ▪ Major fetal congenital malformations that prevent human lactation

#### Interventions

According to the assigned group, early or immediate SSC will take place.

##### Early SSC group (control group)

At birth, the baby will be dried and placed on the abdomen and chest of their mother where thermoregulation maneuvers are applied once cord clamping has been completed. At this time, the baby will placed under the radiant heat lamp in order to complete all newborn adaptation interventions. Once stable, the mother and the baby will proceed with the initiation of SSC for at least 1 h or until the baby has completed the first lactation adequately; SSC will be allowed to continue if the mother expresses a desire to do so. During SSC, the mother and baby will receive monitoring by health personnel. All adaptation interventions, mediate and immediate (Table 3), in the newborn will take place under the radiant heat lamp during the first postnatal hour.

**Table 2:**
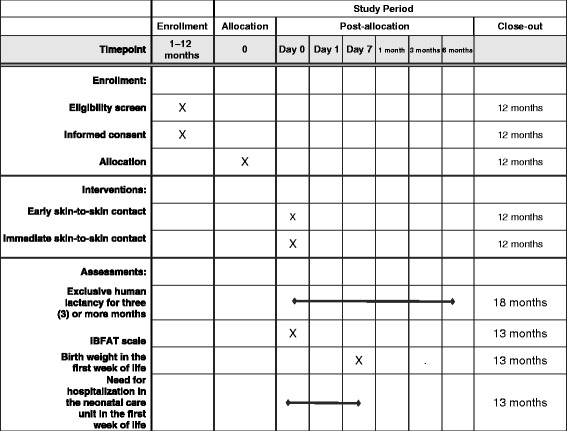
Schedule of enrollment, interventions, and assessments

**Table 3:** Adaptation interventions

Immediate adaptation interventions	Mediate adaptation interventions
• Cleaning of the airways • Drying and stimulating newborn thermoregulation • APGAR rating • Cord clamping • Identifying the newborn • Taking a blood sample from a segment of placental cord for blood group and congenital hypothyroidism screening	• Evaluation of sex and initial physical examination • Application of vitamin K and eye infection prophylaxis • Anthropometric measures

##### Immediate SSC (intervention group)

At birth, the baby will be dried and placed at the mother’s breast where thermoregulation maneuvers will be applied and, once cord clamping has taken place, the baby will be left in SSC with the mother where the immediate neonatal adaptation interventions will take place. Mother and baby will be left in SSC for at least 1 h or until the baby has completed its first lactation properly. Once completed, the baby will be taken to the heat lamp to perform and complete all the newborn mediate adaptation interventions. If the mother expresses the desire to continue in SSC, this will be allowed again after these interventions. During immediate SSC, mother and baby will receive continuous monitoring by the health staff.

#### Outcomes

##### Primary outcome

Exclusive human lactancy for three or more months: exclusive human lactancy is defined as the time in months with human lactation as the only food source and without having received other liquids or solid foods (except drugs and/or vitamins).

##### Secondary outcomes

1. Human lactation capacity in the newborn within the first 24 h of life and prior to discharge using the IBFAT scale (Table 4)
2. Maternal satisfaction with breastfeeding in the first 24 h according to the IBFAT scale (Table 4)
3. Evolution of birth weight in the first week of life
4. The need for hospitalization in the neonatal care unit in the first week of life

##### Instruments and times to assess the results

1. Newborn capacity for human lactation:
  - the Infant Breastfeeding Assessment Tool – IBFAT (newborn human lactancy behavior) within the first 24 h of life and prior to discharge
2. Measurement of the change in weight from birth to the end of the first week of life: this will only be done with a previously calibrated electronic scale, which will take measurements at birth and in the first week of life. The need for admission to the NICU in the first week of life will be assessed during the first follow-up visit in the first the week of life (Additional file 1)
3. Duration of exclusive human lactation: monthly telephone tracking of breastfeeding status will continue for 6 months or until human lactation is no longer the only food source (Additional file 2)

### Study procedures

#### Prior to birth

- ▪ Prior to the start of the study, health personnel (nurses, pediatricians, and obstetricians) will undergo training regarding: SSC after birth in healthy newborns, human breastfeeding concepts (technical, definition of exclusive lactancy and its effective benefits and contraindications), the institutional human lactation protocol and management of the mothers with their newborn baby
- ▪ The mothers who meet the entry criteria will be identified in the obstetrics and gynecology outpatient department at their prenatal appointment. An obstetric history will be conducted by the physician in order to identify risk factors or conditions that may contraindicate the study. Those mothers who meet the criteria for inclusion will be interviewed in order to explain the purpose of the study, its application and other events to be expected. Should they accept the offer of participation, they will sign an informed consent (Additional file 3) and their personal data, telephone number, and email address will be collected for follow-up
- ▪ Those pregnant women who meet the inclusion criteria and sign an informed consent will be classified randomly using opaque envelopes allocated to the intervention or control group. Each envelope will be coded and assigned a study entry number. Prior to delivery, health professionals can determine what the intervention will be and prepare all their personnel to support the mother and child

#### After birth

- ▪ SSC will be applied according to the assigned group (immediate versus early), and measures of immediate and mediate neonatal adaptation will be applied

##### Prior to discharge

Prior to discharge, the IBFAT instrument will be applied to the mother and her infant by the research monitor and/or operational coordinator.

#### Follow-up

- ▪ The first assessment will take place in the first week of life. The mother and the baby will be summoned to the Universidad de La Sabana Clinic and a nursing professional will perform it. This consultation aims to record the birth weight, the effectiveness of human lactation and feeding capacity and the need for hospitalization.
- ▪ Monthly follow-up. This follow-up procedure will take place via monthly telephone calls with the mothers for 6 months, checking their adherence to human lactancy and, in the cases where breastfeeding has been suspended, inquiring about the reasons for this.

### Registration information

An electronic information collection instrument will be created and a paper record of the data collected for the study will also be kept. Periodically (every month), the research team will verify the information in the database with respect to the physical record. To reduce errors in information collection, double entry of information is performed.

The data will be kept in the research office of the Universidad de La Sabana Clinic and only the research group and regulatory personnel (INVIMA) have access to it if, for any reason, it should be required.

### Sample size

24 % babies in Colombia, received exclusive breastfeeding up to 5 months old (9). This was the baseline risk assumed for the sample size calculation [9]. No studies were found that comparing different strategies from SSC; so that estimate the sample size, information 2014 Cochrane meta-analysis was used [3], which compares SCC (regardless of the starting time) with nonintervention. In this meta-analysis, SSC increases exclusive human lactancy in babies of 3 − 6 months of age (RR 1.97, 95 % CI 1.37 to 2.83). With the information of exclusive breastfeeding for Colombia and the effect of SCC﻿, report in the meta-analysis, was estimated ﻿a sample size of 300 newborns. This sample was calculated, two tailed, for a type I error of 5 %, a type II error of 20 %, and an estimated loss rate of 30 %. Will be included 150 newborns per study group.

### Randomization

The 300 newborns will  be randomized using size-6 permuted blocks to ensure that there will be an equal number of participants in the control group and the intervention group. Once the mother signs the informed consent and the entry data for the study has been entered, the operation coordinator of the study will open the opaque envelope, the contents of which determines to which group the mother will be assigned and this information will be recorded immediately in the medical record. The coordinator will verify that the intervention to which the patient was assigned at birth is applied.

### Masking

As to the characteristics of the interventions under evaluation, those who measure the results and who will analyze the data will be blind. To ensure that the blind participant who measures the results will be someone other than the health professional applying SSC, that person will not be in the delivery room and will apply the study measurements to all newborns treated at the clinic without knowledge of whether or not they are study participants.

The person conducting the analysis will be given the database in which the variable that identifies whether the research subject belongs to the control group or the intervention group is found; this will then be coded and the code that identifies them will not be available to the analyst.

### Statistical methods

Descriptive analyses were performed using measures of central tendency (median, average), location (percentiles) and dispersion (standard deviation and ranges) for continuous variables and absolute frequencies and relative for the categories. Bivariate analysis will be performed to determine which variables (evaluation interventions and others) are associated with exclusive lactancy at 6 months. In the continuous variables, the Student’s *t* test will be used for independent samples or the Wilcoxon rank sum test when the assumptions of normality for the *t* test are not fulfilled. The assumption of normality will be evaluated with the Shapiro-Wilk and Kolmogorov-Smirnov tests. Contingency tables will be constructed in the categorical variables, assessing the independence between variables with the chi-square test, or Fisher’s exact test when the assumption of number of cases per cell in the contingency tables, multiplied by two, is not fulfilled. The relative risk (RR) will be calculated as an effects measurement with confidence intervals, adjusted measurements will be calculated using a multivariate Poisson regression model, for adjustment variables with significant results those with biological plausibility will be used in the bivariate analysis. An analysis will be carried out for a two-tailed type I error level of 5 %. The Stata 11 program will be used for the data analysis. An interim analysis will be performed upon submission of half of the expected events (106), setting the limits for early termination of the trial according to the methods proposed by Pampallona and Tsiatis 1994 [21].

The protocol study information will be revised by the Data Monitoring Committee used by the clinic and recruited for the study. This committee will assess the study’s progress, safety data, and critical efficacy endpoints. The DMC reviews unblinded information during the conduct of the study. The information will be periodically revised every 2 weeks.

#### Intention-to-treat analysis

All study participants will be analyzed in the group to which they were originally randomized. Loss to follow-up will be analyzed as explained below:

- ▪ Descriptive analyses﻿ of the lost subjects, will be conducted to determine if they are different from those who continued in the study and whether the losses generated imbalance between the intervention and control groups
- ▪ The effect measure, assuming the worst and best scenario for subjects who were lost to follow-up, will be estimated

## Discussion

SSC at birth has shown benefits in the short and long term for both the mother and the full-term newborn. Although the meta-analyses that have been done have shown the benefits of this technique, multiple differences in the SSC interventions have been identified because criteria such as the initiation or duration of SSC (dose) have not been unified. Studies have been conducted with different schemes (immediate, early, and very early) without clarity about which of the initiation times of SCC from birth provides the greatest benefits during infancy.

Colombia has a malnutrition risk of 11,4 % in the total population for the period 2012-2014, so it is necessary to promote strategies that generate a positive impact on the duration of human lactation, providing support from the clinical setting of humanized delivery which is included in the IAMI strategy (Instituciones Amigas de la Mujer y la Infancia – Friends of Women and Children Institution). Therefore, we propose that the initiation time of SSC in full-term newborns is related to the duration of exclusive human lactation.

Human lactation is simple and freely available, provides optimal nutrition for the baby, facilitating their proper growth and development and, therefore, establishing itself as an effective strategy to help reduce disease in the infant population.

This study aims to determine the effect of SSC initiation times from birth (immediate versus early) in healthy, full-term newborns on the duration of exclusive human lactation at the Universidad de la Sabana Clinic where the newborns were attended. It also evaluates the human feeding capacity of newborns 24 h after birth by applying the IBFAT scale. Thus, the analysis of weight change in the first week of life and the need for hospitalization and admission to the NICU in the first week of life are also included.

### Trials status

This study is not yet open for participant recruitment.

## Appendix

**Table 4:** IBFAT scale (INFANT BREAST-FEEDING ASSESSMENT TOOL)

1. Baby’s state: asleep, drowsy, quiet, alert or crying	
2. Readiness to feed or excitability: the observer should record whether the baby starts to feed effortlessly (3 points), needs mild stimulation to start feeding (2 points), needs vigorous stimulation (1 points) or cannot be awakened (0 points)	
3. Rooting: at the moment the nipple touches the baby’s cheek, he/she turns his/her head towards the nipple, opens his/her mouth and tries to grab the nipple. If the baby is next to the nipple and effectively turns (3 points), if the baby need some help (2 points), if the baby does poorly despite the stimulus (1 points), if the baby does not turn (0 points)	
4. Fixing: the observer records the time from the moment he/she was put on the breast until latching and feeding take place. Latching immediately (3 points), taking 3–10 min (2 points), taking more than 10 min (1 point), not feeding (0 points)	
5. Sucking: the baby does not suck (0 points), sucks poorly (1 points), sucks fairly well but needs help (2 points), sucks very well on one or both breasts (3 points)	
6. Ask the mother if she was feeling: very satisfied, satisfied, a little satisfied or not satisfied. This is then evaluated from 1 to 4; this is not within the scale values	

## Additional files

Additional file 1: Format for data collection in the first face-to-face assessment. (DOCX 12 kb)
Additional file 2: List of data collection in the valuation call. (DOCX 13 kb)
Additional file 3: Informed consent. (DOCX 13 kb)
Additional file 4: Consent for publication. (DOC 24 kb)

## Abbreviations

BSES: Breastfeeding Self-efficacy Scale
IBFAT: Infant Breastfeeding Assessment Tool
MBA: Mother-baby ASSESS tool
NICU: Neonatal Intensive Care Unit
OR: Odds ratio
RR: Relative risk
SSC: Skin-to-skin contact
