# Supplementary material for: The effect of skin-to-skin contact at birth, early versus immediate, on the duration of exclusive human lactancy in full-term newborns treated at the Clínica Universidad de La Sabana: study protocol for a randomized clinical trial
Source: Trials. 2016 Oct 26;17:521. doi: 10.1186/s13063-016-1587-7 (PMC5080719; doi:10.1186/s13063-016-1587-7)
Supplement: Additional file 1: — Format for data collection in the first face-to-face assessment. (DOCX 12 kb) [file 13063_2016_1587_MOESM1_ESM.docx]

**ADDITIONAL FILE 1**

**FORMAT FOR DATA COLLECTION IN THE FIRST FACE-TO-FACE ASSESSMENT**

**FORMAT FOR DATA COLLECTION IN THE FIRST MEDICAL VALUATION LIFE:**

Identification:

- Newborn: ___________
- Mother: _____________

| Age (days) | Weight (grams) |
| --- | --- |
|  |  |

Need for admission to neonatal unit in the first week of life: Yes ___ No ___
