# Supplementary material for: The effect of skin-to-skin contact at birth, early versus immediate, on the duration of exclusive human lactancy in full-term newborns treated at the Clínica Universidad de La Sabana: study protocol for a randomized clinical trial
Source: Trials. 2016 Oct 26;17:521. doi: 10.1186/s13063-016-1587-7 (PMC5080719; doi:10.1186/s13063-016-1587-7)
Supplement: Additional file 2: — List of data collection in the valuation call. (DOCX 13 kb) [file 13063_2016_1587_MOESM2_ESM.docx]

**ADDITIONAL FILE 2**

**LIST OF DATA COLLECTION IN VALUATION CALL**

**GUIDE FOR MONTHLY PHONE CALL TO THE STUDY PARTICIPANT IN THE FIRST**

**6 MONTHS OF LIFE OF**

Good afternoon, my name is ___________ I am researcher at the University of La Sabana working on the SSC study with the prevalent childhood diseases research group.

We are looking for the lady: ___________ to ask her some questions.

If the mother or guardian is identified, the survey will continue.

Has the baby been given?

- Powdered milk? Yes___ No___

If the answer is affirmative ask:

How often has it been given in one day? _______

Is breastfeeding replaced with this? Yes__ No __

Since when has it been given? _______

- Liquid milk? Yes___ No___

If the answer is affirmative ask:

How often has it been given in one day? ____

Is breastfeeding replaced with this?_____

Since when has it been given? ______

- Water? Yes___ No____

If the answer is affirmative ask:

How often has it been given water in one day?

Is breastfeeding replaced with this?

Since when has it been given?

- Fruit juice? Yes___ No___

If the answer is affirmative ask:

How often has it been given in one day?

Is breastfeeding replaced with this?

Since when has it been given?

- Tea, sugar water or herbal tea? Yes___ No___

If the answer is affirmative ask:

How often has it been given in one day?

Is breastfeeding replaced with this?

Since when has it been given?

- Oral rehydration serum? Yes___ No___

If the answer is affirmative ask:

How often has it been given in one day?

Is breastfeeding replaced with this?

Since when has it been given?

- Vitamins? Yes___ No___

If the answer is affirmative ask:

How often has it been given in one day?

Is breastfeeding replaced with this?

Since when has it been given?

- Medication? Yes___ No___

If the answer is affirmative ask:

How often has it been given in one day?

Is breastfeeding replaced with this?

Since when has it been given?

Thank you for your attention. Remember, that this information will be used exclusively for the study.

Have a good day.
