# Supplementary material for: The effect of skin-to-skin contact at birth, early versus immediate, on the duration of exclusive human lactancy in full-term newborns treated at the Clínica Universidad de La Sabana: study protocol for a randomized clinical trial
Source: Trials. 2016 Oct 26;17:521. doi: 10.1186/s13063-016-1587-7 (PMC5080719; doi:10.1186/s13063-016-1587-7)
Supplement: Additional file 3: — Informed consent. (DOCX 13 kb) [file 13063_2016_1587_MOESM3_ESM.docx]

**ADDITIONAL FILE 3**

**INFORMED CONSENT**

**RANDOMIZED BLIND CLINICAL TRIAL OF THE EFFECT OF IMMEDIATE VS. EARLY SSC BIRTH ON THE DURATION OF EXCLUSIVE HUMAN LACTANCY IN FULL-TERM INFANTS CARED FOR IN UNIVERSIDAD DE LA SABANA CLINIC.**

Principal Researcher: Dr. Sergio Agudelo

Contact: sergio.agudelo@clinicaunisabana.edu.co

Introduction

This document aims to provide information related to the research project guidelines **"randomized blind clinical trial of the effect of immediate vs. early SSC birth on the duration of exclusive human lactancy on full-term infants treated at the Universidad de la Sabana Clinic,"** which will be developed by an interdisciplinary team consisting of paediatricians and paediatric residents in training, coordinated by the Department of Paediatrics at the University de la Sabana. After reading and understanding this document, you decide if you accept: participating in the study, accepting the instructions and procedures the team will provide, allow access to the medical history of the mother and newborn and accept that the information collected can be used whilst ensuring that their identities remain protected in investigational studies to be guaranteed in the present or future of the Universidad de la Sabana.

It is important to know some aspects of the study, listed below:

The study starts from the birth process and will continue for the first 6 months of life in which adherence to breastfeeding is to be evaluated.

By agreeing to participate in the study, the participant will cooperate in the supply of data and attend the scheduled visit between the first 3 and 10 days the newborn's life and provide information during the monthly follow-up telephone call for the first 6 months of the infant`s life. There will be no compensation for participating.

As a research group, we guarantee the possibility of the participant withdrawing from the study freely at any time, without the deterioration of the quality of care provided.

The present study seeks to obtain important general knowledge that has an impact on the survival and care of the neonatal population: data that cannot otherwise be obtained.

As explained, I, ______________________________ _______________ identified (a) with the identity document type _________________, No. ____________________, declare that I have received all the information regarding this study and authorize the use of clinical data obtained during the care of my child, ______________________________________, identified ________________ No. ________________________, for purposes of clinical research.

I understand that the purpose of the study is to **evaluate the effect generated by SSC at birth in newborns and its impact on the duration of exclusive human lactancy**. I also declare that I promise to provide health personnel with the information required during the entire follow-up time required for the study. I also declare having been informed that all data will be handled confidentially and that I have the option of refusing to be part of the study or withdraw at any time, knowing that there will be no negative consequence or loss of rights, knowing that I can express all my concerns and that they will be resolved to my satisfaction.

Therefore, I agree to participate voluntarily in this study, the results of which can I access after its completion.

______________________ _________________________

Mother/guardian signature. Researcher signature

________________________                         ______________________

Witness 1 signature            Witness 2 signature
