# Supplementary material for: The effect of skin-to-skin contact at birth, early versus immediate, on the duration of exclusive human lactancy in full-term newborns treated at the Clínica Universidad de La Sabana: study protocol for a randomized clinical trial
Source: Trials. 2016 Oct 26;17:521. doi: 10.1186/s13063-016-1587-7 (PMC5080719; doi:10.1186/s13063-016-1587-7)
Supplement: Additional file 4: — Consent for publication. (DOC 24 kb) [file 13063_2016_1587_MOESM4_ESM.doc]

# Patient’s Consent to Publication

Title of product: **Effect of skin-to-skin contact at birth, early vs. immediate, on the duration of exclusive human lactancy in full-term newborns treated at the Clinica Universidad de La Sabana: study protocol for a randomized clinical trial**

Author/Developer: Sergio Iván Agudelo Pérez

This is to state that I give my full permission for the publication, reproduction, broadcast and other use textual material in all editions of the above-named product and in any other publication (including books, journals, CD-ROMs, online and internet), as well as in any advertising or promotional material for such product or publications.

I declare, in consequence of granting this permission, that I have no claim on ground of breach of confidence or any other ground in any legal system against — (author’s/developer’s name) — and its agents, publishers, successors and assigns in respect of such use of the photograph(s) and textual material (case histories).

I hereby agree to release and discharge (author’s/developer’s name), and any editors or other contributors and their agents, publishers, successors and assigns from any and all claims, demands or causes of action that I may now have or may hereafter have for libel, defamation, invasion of privacy, copyright or moral rights or violation of any other rights arising out of or relating to any use of my image or case history.

Name:

Address:

Signed:

Date: ________________________________
